# Incidence and risk factors for acute shoulder pain after hepatectomy: a nested case-control study

**DOI:** 10.1186/s12871-022-01944-7

**Published:** 2022-12-19

**Authors:** Yuecheng Yang, Yunkui Zhang, Sheng Ling Dai, Lu Wang, Jun Zhang

**Affiliations:** 1 Department of anesthesiology, Fudan University Shanghai Cancer Center, NO 270, Dong-An Road, Shanghai, 200032 People’s Republic of China; 2 Department of hepatic surgery, Fudan University Shanghai Cancer Center, Shanghai, People’s Republic of China

**Keywords:** Shoulder pain, Hepatectomy, Anesthesia, Risk factors

## Abstract

**Background:**

Shoulder pain is commonly reported after hepatic surgery; however, the factors affecting post-hepatectomy shoulder pain remain unclear. This study aimed to determine the incidence and risk factors of shoulder pain after hepatectomy.

**Methods:**

This prospective cohort study recruited 218 patients who underwent hepatic resection at our hospital from June to September 2022. Data were obtained from electronic medical records and follow-up assessments on the second postoperative day. All patients denied chronic pain before surgery. In this cohort study, patients were grouped according to the appearance of shoulder pain. Demographic information and perioperative data were compared between the two groups. The relationship between shoulder pain and independent variables was assessed using univariate binary logistic regression analysis. The potential risk factors were analyzed using multivariable binary logistic regression.

**Results:**

Of the 218 patients enrolled in this cohort study, 91 (41.7%) reported shoulder pain. Patients in the case group were significantly younger than those in the control group (*P* = 0.001). Epidural anesthesia was used more frequently in the case group (*P* = 0.012). Patients over 60 years of age showed a lower incidence of shoulder pain than younger patients (*P* = 0.028). According to multivariable binary logistic regression analysis, advanced age and epidural anesthesia were associated with risk of shoulder pain (advanced age: odds ratio [OR] [95% confidence interval (CI)]: 0.96 [0.94, 0.99], *P* = 0.002; epidural anesthesia: OR [95% CI]: 2.08 [1.18, 3.69], *P* = 0.012).

**Conclusions:**

The incidence of acute shoulder pain after hepatectomy is 41.7%. The application of epidural anesthesia is an independent risk factor for shoulder pain after hepatectomy, whereas advanced age is a protective factor.

## Introduction

Shoulder pain is a common complication after hepatic surgery. The incidence of post-hepatectomy shoulder pain varies between 42 and 75% [1, 2]. In clinical observations, we found that some patients had severe postoperative shoulder pain, while the effect of conventional postoperative analgesia was limited. Recent research has shown that phrenic nerve block alleviates post-hepatectomy shoulder pain [3]. The mechanism of this therapy is that shoulder pain is a referred pain via the phrenic nerve. A previous cohort study showed that shoulder pain occurred more frequently in patients receiving epidural anesthesia while undergoing hepatectomy [2]. Pneumoperitoneum also plays a role in shoulder pain after laparoscopic cholecystectomy [4].

Overall, there is a paucity of studies on shoulder pain after hepatectomy. The only previous study on post-hepatectomy shoulder pain was a retrospective cohort study. Patients were grouped according to the type of anesthesia. In addition, the sample size in this study was relatively small. Multiple factors are likely associated with shoulder pain after hepatectomy. Identifying the risk factors contributes to the prevention of shoulder pain. To the best of our knowledge, no study with a large sample size has been conducted to determine the possible risk factors for shoulder pain after hepatic surgery.

To fill the gap, in the current study, we hypothesized that some risk factors may increase the incidence of shoulder pain. To this end, we conducted a nested case-control study of hospitalized surgical patients.

## Methods

### Study design

The current study was a case-control study nested in a prospective observational cohort. Because of the observational nature and all data collected from the medical records, the study was approved by the Ethics Committee of the Shanghai Cancer Center (Ethics Approval Number: 1612167–18), and written informed consent was waived.

### Study population

Patients were recruited consecutively at the Department of Hepatic Surgery at Shanghai Cancer Center, Fudan University, from June 2022 to September 2022. The inclusion criteria were as follows: (1) American Society of Anesthesiologists score of I to III; (2) patients who underwent hepatectomies under anesthesia; and (3) patients denied having chronic pain before surgery (based on medical records).

The exclusion criteria were as follows: (1) multivisceral resection, (2) history of shoulder pain, and (3) patients whose surgeries were converted to open surgery after a laparoscopic attempt.

### Data collection

Biometrics (including age, sex, height, and weight) and medical data (such as hypertension, diabetes, surgical history, surgery duration, consumption of Sufentanil, type of anesthesia, overall pain scale, and surgical approaches) were collected from the electronic medical record system. Postoperative data were collected within 40–48 h after surgery. When the patient-controlled analgesia devices were taken from the patients, they were asked whether they experienced shoulder pain.

### Perioperative procedures

All patients underwent standard monitoring. The exclusion criteria for epidural anesthesia were as follows: (1) patients who refused epidural anesthesia, (2) elderly patients who could not maintain an epidural position, and (3) patients who could not cooperate because of language barriers. All epidural anesthesia procedures were performed by the attending anesthesiologists (experience of more than 500 cases). For patients receiving epidural anesthesia, a test dose of 3 mL of 1% Lidocaine was administered after the epidural catheter was inserted. Epidural analgesia was intermittently administered using 0.25% Ropivacaine. The spinal level of epidural anesthesia was tested using wet cotton balls. The spinal level of epidural anesthesia was at T6–T10. None of the patients complained of dyspnea. General anesthesia was induced with Propofol, Rocuronium, and Sufentanil. Desflurane, or Sevoflurane, Propofol, and Remifentanil were used to maintain anesthesia. An intermittent bolus of Sufentanil was used for intraoperative analgesia. Postoperative analgesia was achieved using epidural patient-controlled analgesia in patients receiving epidural anesthesia. According to our institution’s routine protocol, the epidural analgesia pump was set to a volume of 200 mL, containing 300 mg of Ropivacaine and 100 μg of Sufentanil. The patient-controlled device was programmed to deliver a 4 mL bolus with a lockout interval of 20 min and a background infusion of 4 mL/h. For patients receiving general anesthesia alone, intravenous patient-controlled analgesia was administered. The intravenous analgesia pump contained Sufentanil 100μg and Flurbiprofen Axetil 150 mg (or Ketorolac Tromethamine 90–105 mg). Intraoperative and postoperative intravenous analgesia were independently determined by the attending anesthesiologist.

The patient was placed in the supine position if the expected surgical site was the left lobe of the liver. When the right liver lobe was expected to be removed, for a good visual field during the surgery, a padded cushion was placed under the patient’s right waist. In laparoscopic surgeries, the pneumoperitoneum pressure was set at 12–15 mmHg.

All patients routinely underwent overall pain scale assessments before leaving the post-anesthesia care unit (PACU). Pain intensity was assessed using a numerical rating scale (NRS). Additional intravenous Sufentanil (3–5 μg) or epidural Ropivacaine (0.25%, 3–5 ml) was administered if necessary.

### Cases and controls

In this cohort, cases were patients who reported shoulder pain regardless of type (such as dull pain and aching pain) and severity within 48 h after the surgery. Shoulder pain data were recorded as either left, right, or bilateral. Controls included patients who did not report any pain around the shoulder.

### Sample size calculation

The sample size was calculated using the PASS (Version 2021, Kaysville, Utah, USA). Statistical significance was set at 0.05, with a power of 90%. The ratio of cases to controls was set at 1:1.5 (based on a previous study, the incidence of shoulder pain was approximately 40%). The odds ratio was set at 2.5, and the proportion in the control group was set at 0.4. The sample size was calculated using the Z-test. Therefore, the calculated minimum total sample size was 205.

### Statistical analysis

The Kolmogorov–Smirnov test was used to determine whether the meteorological data followed a normal distribution. Normally distributed continuous data are expressed as mean ± standard deviation. The differences in measurement data (age, height, body mass index [BMI], and surgical duration) between the case and control groups were calculated using *t*-test. Categorical variables (sex, comorbidities, use of laparoscopy, use of epidural anesthesia, and surgical history) between the two groups were compared using the chi-square test.

Univariate binary logistic regression was used to determine the possible risk factors (including age, sex, height, BMI, surgical duration, use of epidural anesthesia, use of laparoscopy, comorbidities, and surgical history) for shoulder pain. Variables with *P* < 0.2 in the univariate binary logistic regression model were included in the multivariate binary logistic regression model. The odds ratio (OR) for shoulder pain is presented with a 95% confidence interval (CI). The significance level was set at 0.05. All analyses were performed using the Statistical Product and Service Solutions (SPSS Version 22.0, IBM, USA).

## Results

During the study period, 218 patients underwent hepatectomies. A total of 91 patients reported postoperative shoulder pain. Right shoulder pain occurred in 63 patients, whereas left shoulder pain occurred in 20 patients. Furthermore, eight patients reported bilateral shoulder pain.

The overall pain score of all patients in the PACU ranged from 0 to 2 (NRS). Seven patients required additional Sufentanil (3–5 μg). All patients received general anesthesia alone.

The patient characteristics are shown in Table 1. Patients in the case group were significantly younger than those in the control group (*P* = 0.001). Epidural anesthesia was used more frequently in the case group than in the control group (*P* = 0.012). The mean consumption of Sufentanil was significantly more in the control group (*P* = 0.005). There were no statistical differences in sex, height, BMI, surgical duration, application of laparoscopes, comorbidities, surgical history, and overall pain scale between the two groups.

**Table 1:** Demographic and perioperative data

Variables	Controls Patients	Cases Patients	*P* Value
Number of Patients	127	91	/
Gender (Male)	87 (68.5%)	56 (61.5%)	0.286
Age (years)	61.1 ± 11.7	55.7 ± 12.9	0.001
Height (cm)	166.2 ± 7.5	166.3 ± 6.9	0.93
BMI (kg/m^2^)	23.7 ± 3.8	23.2 ± 3.7	0.30
Hypertension	28 (22%)	16 (17.6%)	0.49
Diabetes	11 (8.7%)	8 (8.8%)	1.0
Surgical History	73 (57.5%)	52 (57.1%)	1.0
Laparoscopic/ Open	44/83	35/56	0.56
Epidural Anesthesia	62 (48.8%)	60 (65.9%)	0.012
Sufentanil (ug)	34.9 ± 9.2	31.4 ± 8.3	0.005
Surgical Duration (min)	118.8 ± 51.0	120.9 ± 57.1	0.78
Overall Pain Scale	1.34 ± 1.04	1.62 ± 1.25	0.09

Enumeration data were presented as the cases (percentage). Sufentanil: Intraoperative consumption of Sufentanil. Overall Pain Scale: Assessed on the second day after surgery using the numerical rating scale (NRS)

In the original cohort, the consumption of Sufentanil (μg) was significantly less in patients receiving epidural anesthesia (*n* = 122) than in patients receiving general anesthesia alone (*n* = 96) (27.1 ± 4.3 VS 41.5 ± 6.6, *P* < 0.001).

**Fig. 1:**
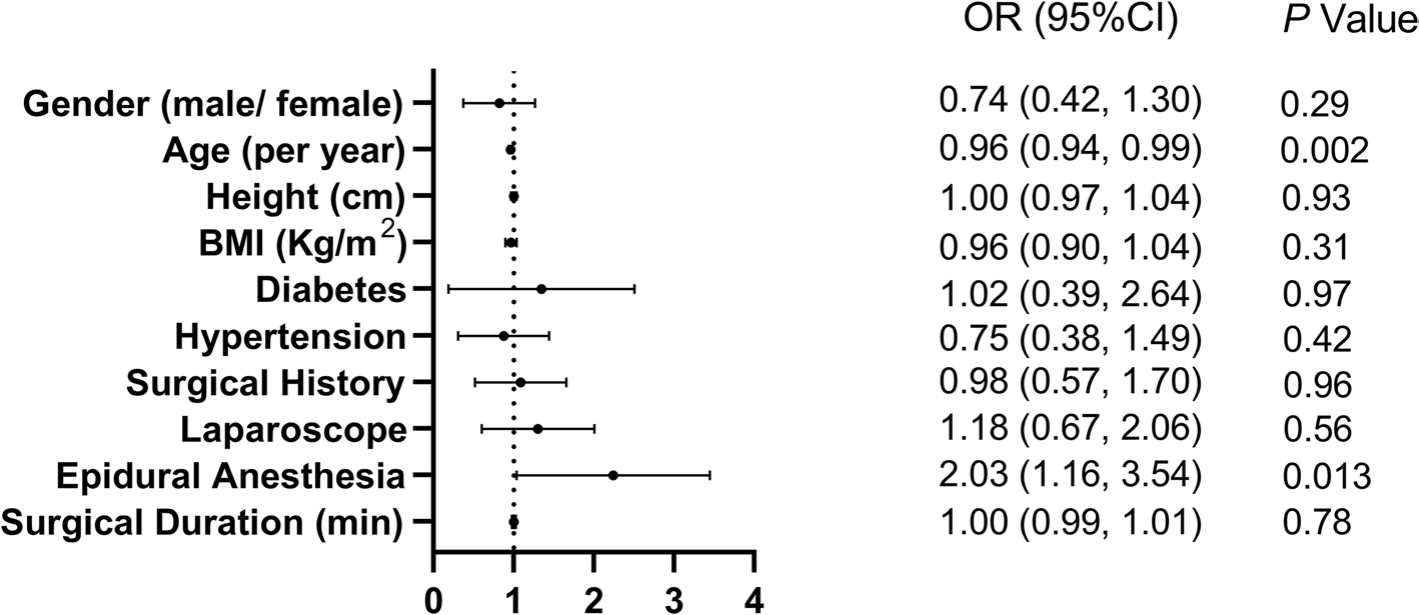
Univariate binary logistic regression for shoulder pain. Variables were calculated using the univariate binary logistic regression model. The effect of each variable on shoulder pain was presented in odds ratio and 95% confidence interval. Odds ratio greater than 1 represented increased odds for shoulder pain. *P* values were calculated using univariate binary logistic regression analysis

All patients were divided into two groups according to their age. We used a cutoff age of 60 years. The incidence of shoulder pain in the younger age group was significantly higher than that in the older age group (59 [48.4%] vs 32 [33.3%], *P* = 0.028). The effects of demographic and perioperative data on shoulder pain were analyzed using univariate binary logistic regression (Fig. 1). Advanced age was associated with decreased odds of postoperative shoulder pain (OR [95% CI], 0.96 [0.94, 0.99]; *P* = 0.002). The application of epidural anesthesia was associated with increased odds of shoulder pain (OR [95% CI]: 2.03 [1.16, 3.54], *P* = 0.013). The effects of the other variables on shoulder pain were not significant.

**Fig. 2:**
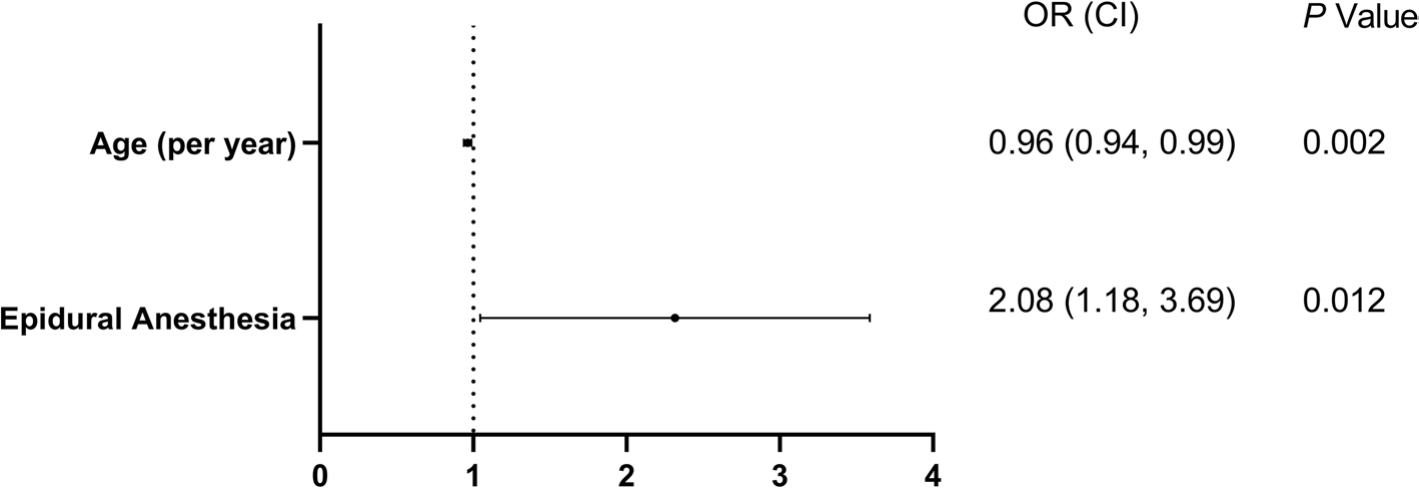
Multivariate logistic regression for shoulder pain. Based on the result of univariate binary logistic regression analysis, age and epidural anesthesia were included in a multivariate logistic regression model. Odds ratio greater than 1 represented increased odds for shoulder pain. *P* values were calculated by multivariable binary logistic regression analysis

Based on the results of the comparison of the two groups and the univariate binary logistic regression analysis, age and application of epidural anesthesia were included in a multivariate binary logistic regression analysis (Fig. 2). Both variables were significantly associated with post-hepatectomy shoulder pain (advanced age [per year]: OR [95% CI], 0.96 [0.94, 0.99]; *P* = 0.002; epidural anesthesia: OR [95% CI], 2.08 [1.18, 3.69]; *P* = 0.012).

## Discussion

This observational study investigated the incidence and risk factors of shoulder pain after hepatectomy. The total incidence of shoulder pain was 41.7%, and epidural anesthesia increased the odds of shoulder pain. By contrast, advanced age decreased the risk of shoulder pain.

The total incidence of shoulder pain was consistent with that reported in a previous study [2]. Owing to the larger sample size, we further found that shoulder pain occurred only on the left side in a small number of patients. A possible explanation is that a large portion of the liver is located on the right side of the body. Hence, intraoperative manipulation is more likely to stretch the right side of the diaphragm. Although the influence of the intraoperative position on shoulder pain could not be excluded, we found that shoulder pain was also common in patients in the supine position during surgery. This suggests that myofascial pain caused by intraoperative posture is not the primary cause of shoulder pain [2]. We inferred that operative manipulation around the liver irritated the diaphragm, resulting in shoulder pain.

Younger age has been recognized as a predictive factor for postoperative pain [5]. Younger individuals have a higher risk of moderate-to-intense pain after surgery [6]. The overall mean difference in pain intensity between the young and the old is 1.5 points (a total of 10) [7]. However, the effect of age on shoulder pain remains controversial. Wada et al. reported that younger age was a risk factor for gynecological laparoscopy [8]. By contrast, Nutchanart et al. found that age was not associated with the incidence of shoulder pain after thoracic surgery [9]. In this study, we found that younger patients had a higher risk of shoulder pain after hepatectomy. The possible mechanisms are age-related anatomical, physiological, and biochemical changes, as well as intrinsic plasticity of somatosensory pathways in old patients [10]. The effect of age on the logistic regression analysis was minimal because the effect was based on every additional year of age.

Shoulder pain occurs more frequently in patients receiving epidural anesthesia than in those receiving general anesthesia alone [2]. In this retrospective study, patients were grouped based on the type of anesthesia. Consistent with previous findings, we found that patients with shoulder pain more frequently had received epidural anesthesia. In addition, we confirmed that epidural anesthesia is an independent risk factor. Neural transmission routes cannot be blocked by epidural anesthesia [11]. Hanna et al. found that when the epidural catheter was placed at the T5 level or higher, the incidence of postoperative shoulder pain decreased [12]. This result might be associated with the spread of the local anesthetic in the epidural space. Normally, a local anesthetic applied at the T7–8 level cannot block the phrenic nerve during epidural anesthesia. The use of epidural anesthesia reduces the amount of intraoperative analgesics, especially opioids. Clinically, we tend to offer a greater variety of analgesics to patients receiving general anesthesia alone. In this study, we also found that patients without shoulder pain had received higher amounts of Sufentanil than those with shoulder pain. Hence, epidural anesthesia may not directly lead to shoulder pain. Insufficient epidural anesthesia-related systemic analgesics may increase the risk of shoulder pain. Despite effective epidural analgesia in the abdominal area, shoulder pain persisted. Although shoulder pain caused by the phrenic nerve is refractory, non-opioid medications still provided partial pain relief [13]. Furthermore, postoperative intravenous analgesia (opioids and nonsteroidal anti-inflammatory drugs) may be associated with a reduced incidence of shoulder pain. Further randomized controlled trials are required to evaluate the efficacy of these analgesics.

Sex differences in postoperative pain are well known. However, previous studies focusing on sex differences have yielded inconsistent results. Hans et al. found that females reported a 0.29 point (a total of 10) higher than males [7]. By contrast, another systematic review suggested that sex is not a predictor of postoperative pain [5]. The mechanisms of sex differences in pain were as follows: first, temporal summation of pain intensity and unpleasantness ratings were more pronounced in females than in males [14]. Second, women are more likely to overestimate pain intensity owing to cultural differences [15]. Third, possible confounders, especially anxiety and stress, may be actual contributors to increased pain [5, 15]. Although the female sex being a risk factor for postoperative pain was controversial, the sex difference in pain intensity was minimal. In this study, males had relatively low odds of shoulder pain. However, the differences were not significant. The possible differences and mechanisms between sexes may require further study with larger sample sizes. Possible confounders, especially psychological factors, should be evaluated preoperatively.

It is widely acknowledged that laparoscope-assisted techniques induce shoulder pain during gynecological operations and cholecystectomy [4, 16]. Shoulder pain is caused by diaphragmatic irritation caused by insufflation gas. The use of a lower-pressure technique reduces the incidence of shoulder pain [4]. It was also found that residual CO_2_ is involved in the maintenance of shoulder pain [17]. In this study, patients undergoing open surgery also experienced shoulder pain, suggesting that laparoscopy is not the only factor. Surprisingly, the use of laparoscopes did not increase the odds of shoulder pain. The possible explanations are as follows: (1) compared with laparoscopic surgery, open surgery requires a more sufficient separation of the liver, which may enhance the irritation of the diaphragm; and (2) because of the laparoscopic field and the concern regarding bleeding, laparoscopic procedures are usually gentler, especially during separation of tissues.

Surgical duration was not associated with the incidence of shoulder pain. Previous studies reported that surgical duration was a risk factor for thoracic and gynecological laparoscopic surgery [8 9]. However, we failed to replicate the findings of this study. This intriguing result could be attributed to the influence of both the surgical procedures and pneumoperitoneum on the diaphragm. Typically, the surgical duration is an important manifestation of surgical difficulty. This result suggests that the extent of resection does not contribute to the risk of shoulder pain.

A recent study also emphasized that patients with a lower BMI showed a higher incidence of shoulder pain after laparoscopic surgery for infertility [18]. By contrast, another study found that BMI was higher in patients with postoperative shoulder pain [12]. In the present study, we found that BMI was not associated with the risk of shoulder pain.

This study had some limitations. First, the number of possible risk factors was relatively limited mainly because the range of surgical resection was not included in this study. As hepatectomy is a non-standardized surgical procedure because of the size and site of the tumor, a variety of procedures cannot be simply classified. The liver is anatomically close to the diaphragm; therefore, the influence of surgical procedures on shoulder pain should not be ignored. Further studies with larger cohorts are required to investigate the impact of surgical site on shoulder pain. Second, medication administration and use of epidural anesthesia during anesthesia were unstandardized. Third, the surgical approaches were determined by the surgeons and patients. As a result, most patients with intra-abdominal adhesions undergo open surgery. Although the effect of intra-abdominal adhesion on shoulder pain is unknown, it could also increase the risk of bias in this study.

## Conclusion

The incidence of shoulder pain after hepatectomy is nearly two-fifths. This study demonstrated that epidural anesthesia was an independent risk factor for shoulder pain after hepatectomy, whereas advanced age was a protective factor. Our findings may contribute to the prevention of post-hepatectomy shoulder pain. Despite effective epidural analgesia, systemic analgesia remains necessary to cope with the alternative pathway of shoulder pain. The effect of anesthetic management on shoulder pain requires further prospective studies.

## Data Availability

The datasets used during the current study are available from the corresponding author on reasonable request.

## Abbreviations

BMI: Body mass index
CI: Confidence interval
NRS: Numerical rating scale
OR: Odds ratio
PACU: Post-anesthesia care unit
